# Evidences of Poisoning by Prussic Acid

**Published:** 1849-01

**Authors:** 


*Evidences of Poisoning by Prussic Acid.*—Dr. W. Wright, of M'Gill College, British America, deduces the following inferences upon the different points connected with the above subject:—

I. *As to the State of the Eyes.*—1. That there is no constant state of the eyes. 2. That the eye-balls are generally prominent, glistening, and bright; pupils dilated, and more or less insensible.

II. *State of the Countenance.*—1. The countenance may retain its natural appearance. 2. It may be pale and calm, as in sleep. 3. It may be livid, turgid, and distorted, with foam issuing from the mouth. 4. That this state is analogous to that observed in epilepsy, while that immediately preceding resembles asphyxia from carbonic acid.

III. *State of the Respiration.*—1. Respiration is variously affected, and may be (*a*) noiseless and tranquil, as in sleep; (*b*) one or two gasps or inspirations; (*c*) noisy and stertorous; (*d*) convulsive and catching; (*e*) slow and laborious. 2. That restoratives cause deep inspirations. 3. That owing to the above discrepancies, we cannot predict with certainty, that any specific change will be induced in the respiration by prussic acid.

IV. *State of the Pulse.*—1. That our knowledge of the effects of a fatal dose of prussic acid on the pulse is very imperfect, all that can be said is that it renders the pulse imperceptible.

V. *Consciousness.*—1. After a fatal dose, consciousness, reason, and intelligence, are retained for an interval of variable duration. 2. That this interval is usually short, and is succeeded by complete insensibility. 3. That the absence of delirium and mental hallucination are negative characteristics.

VI. *Acts of Volition.*—1. That a person after taking a fatal dose of prussic acid may retain, for a period, command over the voluntary muscles, sufficient to perform various and complicated actions.—2. That the duration of this period cannot be fixed. 3. That the powers of speech may not be immediately annihilated by a fatal dose, and it is not unlikely that they may be enjoyed as long as power and command over the voluntary muscles, generally, are maintained. 4. That, as in one instance, a rational answer was spoken by the victim, to a question three minutes after he had swallowed the fatal dose, so, *a fortiori*, the period, during which consciousness, volition, and motion are exertible, might be, possibly, of three minutes duration. 5. That, with this knowledge, we should be prepared to make allowances for the fulfilment of different intricate performances, in the interval of consciousness and muscular control, which follows the taking of poison. 6. Convulsions occurred in none of the cases cited above, it is, therefore, erroneous to connect them exclusively with those of slow death, or with those where subjects enjoyed more or less muscular control prior to death.

VII. *Of Sensation.*—1. That, with loss of consciousness, there is loss of sensation. 2. Prior to the occurrence of which, it is not unlikely that the feelings are not of a disagreeable nature. 3. That prussic acid produces some particular effect on the special senses, as great bitterness of taste, and loud ringing in the ears. 4. That should restoratives be successful, sensation returns, accompanied with feelings more or less perverted, and varying from those of slight uneasiness to those of great agony. 5. That the property which prussic acid possesses of obliterating sensation, classes it among the anæsthetic agents.

VIII. *Of Motion.*—1. That the most important varieties of involuntary motion, observable after fatal doses of prussic acid have been swallowed, are—first, tonic spasm or tetanic rigidity ; and, secondly, clonic spasms or convulsions, (epilepsy.) 2. That probably the tonic result from a major dose and greater susceptibility ; and the clonic from a minor dose and less susceptibility. 3. That both, more especially from a maximum dose, may be absent. 4. That death is least rapid in cases of convulsions. 5. That convulsions may be succeeded by rigidity. 6. That rigidity may occur without convulsions, and that it usually appears on the departure of convulsions. 7. That the absence of convulsions is denoted by placidity of the countenance, non-clenching of the hands, natural posture, want of derangement of the clothes, and other marks of struggling. 8. The results from experiment on animals as regards convulsions, are not to be expected in the human subject, in whom convulsions are not constant but occasional symptoms.

IX. *Of the Odour.*—1. That the odour is not always present in the breath in cases of poisoning by prussic acid. 2. That if present, it may be questionable from—first, its weakness, which may be due to many causes ; secondly, its being masked by other odours ; and thirdly, from discords in the opinions of those testing it.

X. *Of the Shriek.*—1. That in man the absence of the shriek is the rule, its presence the exception. 2. It is not improbable that loud gasping inspirations have been mistaken for it. 3. That when it does occur, it is most likely a simple expression of terror, wherefore it might be present only in homicidal or accidental cases. 4. That it would be likely to occur in cases of epilepsy and convulsions.

XI. *The evacuation of the Rectum and Bladder* is an accidental, rather than an essential symptom.—*British American Journal.*

